# Lockdown as an Intervention Measure to Mitigate the Spread of COVID-19: a modeling study

**DOI:** 10.1590/0037-8682-0417-2020

**Published:** 2020-10-21

**Authors:** Aédson Nascimento Góis, Estevão Esmi Laureano, David da Silva Santos, Daniel Eduardo Sánchez, Luiz Fernando Souza, Rita de Cássia Almeida Vieira, Jussiely Cunha Oliveira, Eduesley Santana-Santos

**Affiliations:** 1 Universidade Estadual de Campinas, Instituto de Matemática, Estatística e Computação Científica, Campinas, SP, Brasil.; 2 Instituto Federal de Educação, Ciência e Tecnologia de Alagoas, Coordenação de Formação Geral, Campus Piranhas, Piranhas, AL, Brasil.; 3 Universidade Tiradentes, Departamento de Enfermagem, Aracaju, SE, Brasil.; 4 Universidad Austral de Chile, Centro de Docencia de Ciencias Básicas para Ingeniería, Valdivia, Chile.; 5 Universidade de São Paulo, Escola de Enfermagem, São Paulo, SP, Brasil.; 6 Universidade Federal de Sergipe, Programa de Pós-Graduação em Enfermagem, Aracaju, SE, Brasil.

**Keywords:** COVID-19, Coronavirus infection, Social isolation, Epidemiology

## Abstract

**INTRODUCTION:**

This work aims to develop a biomathematical transmission model of COVID-19, in the State of Sergipe, Brazil, to estimate the distribution of cases over time and project the impact on the spread of the epidemic outbreak due to interventions and control measures over the local population.

**METHODS:**

This is an epidemiological mathematical modeling study conducted to analyze the dynamics of the accumulated cases of COVID-19, which used a logistic growth model that adds a term of withdrawal of individuals as a control measure. Three possible COVID-19 propagation scenarios were simulated based on three different rates of withdrawal of individuals. They were adjusted with real data of the infected and measures of control over the population.

**RESULTS:**

The lockdown would be the best scenario, with a lower incidence of infected people, when compared to the other measures. The number of infected people would grow slowly over the months, and the number of symptomatic individuals in this scenario would be 40,265 cases. We noticed that the State of Sergipe is still in the initial stage of the disease in the scenarios. It was possible to observe that the peak of cases and the equilibrium, in the current situation of social isolation, will occur when reaching the new support capacity, at the end of August in approximately 1,171,353 infected individuals.

**CONCLUSIONS:**

We established that lockdown is the intervention with the highest ability to mitigate the spread of the virus among the population.

## INTRODUCTION

The infection caused by the new coronavirus, responsible for causing respiratory infections of zoonotic viral origin, has been the subject of constant research on its dissemination and potential for contagion among the population. The first reported case occurred in late December 2019, still of unknown etiology in the city of Wuhan, China. On February 11, 2020, the World Health Organization (WHO) recognized the virus as SARS-CoV-2, responsible for causing the disease COVID-19, which quickly spread around the world and became a pandemic with high dissemination and contagion level. In Brazil, the first case was dated February 26, 2020, in the southeastern part of the country, in the State of São Paulo, and quickly spread to several states. On March 6, the first case ever was recorded in the northeast of the country, in the State of Bahia. On March 15, the first case was confirmed in Aracaju, the capital of the State of Sergipe, warning of the high speed of the spread of the disease^1^ ^-^ ^3^. 

Until this moment, it is known that the primary means of transmission are droplets and aerosols (droplet nuclei) from the air and respiratory tracts and can also be transmitted by indirect contact through the surfaces or objects on which the infected come into contact^4^. When a person is infected, some characteristic signs and symptoms similar to a flu-like syndrome are presented, such as fever, tiredness, cough, nasal congestion, runny nose, and/or sore throat^5^. Cases considered severe evolve to respiratory distress syndrome^6^. In cases where individuals are infected and symptomatic, a study reveals that approximately 20% will need hospitalizations, aiming at improving their clinical condition. For 5% of these, due to hemodynamic instability, there is a need for treatment in an intensive care unit (ICU)^7^. For such patients, the worst prognosis is directly related to the presence of comorbidities, such as advanced age, immunosuppression, diabetes mellitus, respiratory and cardiac diseases^8^. 

While a vaccine to combat COVID-19 is yet to be developed, the authorities have established the use of a mask, social distancing, social isolation, and quarantine as forms of containment of COVID-19^9^ due to the number of ICU beds (0,7% of the total in the country) needed to support the most severely ill patients. Additionally, studies are required to contribute to the analysis of estimates regarding the distribution of cases in the States as well as possible impacts of the epidemic given the control measures adopted. 

In the current scenario in which strategies to contain or minimize the spread of the virus are essential, the objective of this study was to develop a biomathematical transmission model of COVID-19 in the State of Sergipe, Brazil. Wherein, using numerical simulation computational tools to estimate the distribution of cases over time and to assess the impact of interventions (control measures on isolation and/or social distance) on the size and speed of growth of the epidemic outbreak.

## METHODS

This is an epidemiological mathematical modeling study. The epidemiological data used were from people infected in the State of Sergipe, in northeastern Brazil, made available by the Ministry of Health^10^. For that, a logistic growth model was used that adds a withdrawal term for individuals as a control measure^11^. 

The logistic growth model was chosen in relation to the usual compartmental models due to the analytical advantages that will be presented below. Although it is possible to obtain a numerical solution for the number of accumulated cases via computer simulations and parameter adjustments, the associated uncertainty is high since many parameters are used.

The following Initial Value Problem (IVP) satisfactorily models our problem:

$$\frac{dI\left(t\right)}{dt}=rI\left(t\right)\left(1-\frac{I\left(t\right)}{K}\right)-pI\left(t\right),\;I\left(0\right)=I_{0}$$

In this equation *I*(t) represents the number of individuals infected by the coronavirus at time *t*, given in days, *r* refers to the rate of contagion with free mobility between people, *K* is the carrying capacity of the environment and *p*; the percentage of individuals (infected or not) removed from social life via isolation. We can still rewrite the differential equation as:

$$\frac{dI(t)}{dt}=\left(r-p\right)I(t)\left(1-\frac{I(t)}{\frac{r-p}{r}K}\right)$$

This way, 0 ≤ p ≤ r will be considered because the withdrawal rate *p = 0* establishes no control measure applied, and *p = r* means maximum control measure capacity achieved.

In a classic logistical model, the carrying capacity may be conceptualized as the maximum number of individuals that an ecosystem can sustainably support^11^. Thus, for our model, *K* refers to the maximum number of infected individuals to reach in a specific region. Given the possibility of contagion most individuals, except for interventions, we used the population of the State of Sergipe, *K* = 2,298,696, as an initial support capacity^12^.

It is possible to notice that *p* is established as the factor responsible for the control of *I*. Therefore if *p = 0* we will have the case of the classic logistic model in which growth is free and equilibrium over time will occur in *I = K*, that is, without any control measures. In the past, if *p ≠ 0*, with *p <r,* we observe that the new support capacity will be *K. (r - p)/r*. Consequently, it is for this new value that function I will reach its equilibrium. Also note that the higher the *p-*value, the lower the new balance.

From the data made available by the Ministry of Health from March 15 to June 2, it was possible to adjust the parameters so that the epidemic curve was as close to the curve as the actual data collected. Therefore, data for the contagion rate were used until March 24, date of government decree No. 40.567, even when there was a free circulation of the population, being then optimized to *r* = 0.1885. This aspect represents the scenario of the free movement of people having p = 0.

For the social isolation scenario, data were used until June 2. The removal of the portion of the infected population and the groups considered at risk (elderly and people with comorbidities) were considered. After optimized data, maintaining the value of r = 0.1888, the value p = 0.0925 was obtained. The data was generated from the correspondent period, March 24, assuming that society adheres to the social isolation measures requested. 

The last scenario studied is related to total isolation, also called community containment or blockade. In this scenario, there would be a ban on the movement of people outside their houses unless in cases of extreme need. Therefore, it was used *p* = 0.1225, which is 65% of *r -* keeping people who provide essential services free from isolation.

Based on the PVI solution, it was possible to obtain an expression that relates the portion of the population to be isolated via preventive strategies and the time required for the inflection in the infected curve to happen. 

Namely,

$$t=\frac{\ln\left(\frac{r-p}{r}K-1\right)}{\left(r-p\right)}$$

Thus, it is justified to use the parameters r and p described above to obtain a more adequate carrying capacity.

It is worth to mention that the distancing from the inflection point also results in a decrease in the height of the epidemic peak. Given that the proposed model is continuous with respect to the parameters, we have numerical stability of our method in the sense that small disturbances in the settings produce close solutions quantitatively and qualitatively.

We plotted the graphs using GNU Octave, free software with computational language that allows us to solve differential equations numerically like our model.

## RESULTS

**FIGURE 1:**
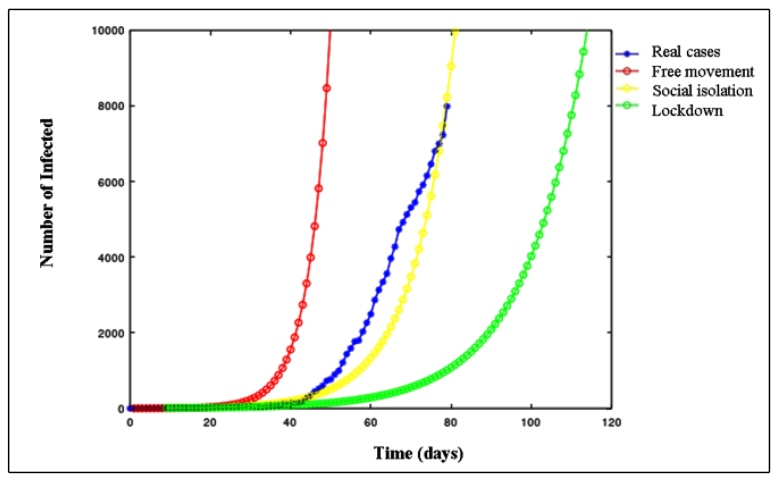
Evolution of the pandemic in Sergipe from March 15 to June 2.

Figure 1 shows the decent number of confirmed infection cases until June 2 (t = 79), the blue curve. On that date, the number of infected was 7989 individuals. Further, it is possible to observe the evolution of infection day by day in four different scenarios: (1) the red curve represents the isolation-free scenario; (2) the yellow curve refers to the growth of the infection based on the measure of social isolation, instituted in Sergipe; (3) the green curve estimates the situation in the State if a lockdown was decreed and; (4) the blue curve represents the real cases.

It is possible to notice the accelerated growth of contagion in the free scenario that at the end of April, the number of infected people would be 8000. However, the yellow curve grows much more slowly, registering this number only in early June. However, if the lockdown had been implemented since March 24, the number of people infected with COVID-19 in early June would have been only 80 cases.

**FIGURE 2:**
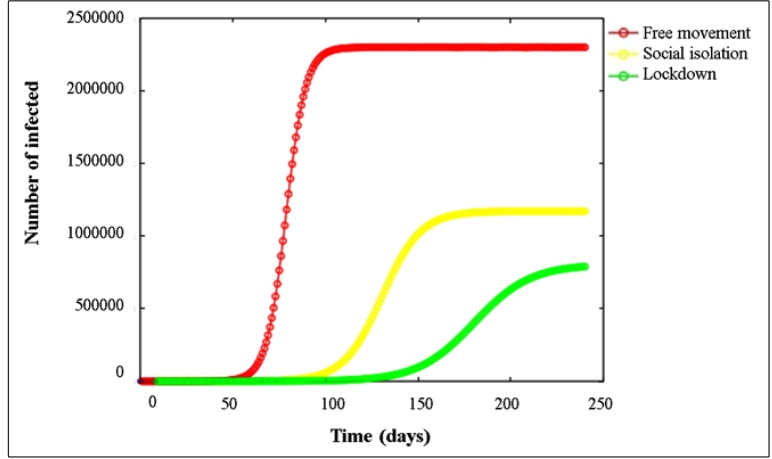
Projection of the evolution of COVID-19 in the State of Sergipe until November 15 in three different situations.

From this perspective, it is still possible to more accurately analyze the interference of isolation measures in containing the contagion of COVID-19 when the evolution of the number of infected people is projected in the next five months. Figure 2 projects the number of infected individuals until the first half of November 2020. Attention is drawn especially to the green curve, which represents the scenario in the hypothesis of total isolation (allowing only the mobility of professionals from essential services) and complete closure of trade.

Observing the epidemic curves, it is clear that the State of Sergipe is still in the initial stage of the disease in any of the scenarios. Thus, the State is heading for the moment when there will be a drastically accelerated growth if social isolation is interrupted (red curve). It is still possible to observe that peak cases and equilibrium, in the current scenario of social isolation (yellow curve), will occur when the new support capacity is reached, at the end of August the formula could estimate this:

$$\frac{K.(r\;-\;p)}{r}$$

In approximately 1,171,353 infected individuals.

The extreme measure of total isolation, or lockdown, would be the best scenario with the lowest incidence of infected people when compared to the other measures. The number of infected people would grow slowly over the months, reaching approximately 154,250 in the first half of August, as seen on the green curve. In using the same formula to calculate the new stabilization, a maximum of approximately 805,305 cases would be observed in November. Taking into account the small portion of the population of Sergipe that is being tested, we estimate that the number of symptomatic individuals in this scenario would be 40,265 cases.

## DISCUSSION

The main finding of this study was that the rate of contagion modeled could be quite accelerated if official isolation recommendations are not met, whether partial or total (red curve in Figure 2). Our findings suggest that state control of outbreaks is viable with a combination of the proposed intervention measures that involve, in addition to the hygiene actions recommended by WHO, the adoption of lockdown (green curve in Figure 2).

The first case of COVID-19 in Sergipe confirmed on March 15, 2020, was a woman that lives in the capital Aracaju, aged 36, and who had returned from Spain. By personal decision, she was isolated for a few days at her residence, and when she decided to undergo tests, her infection was confirmed. Since the confirmation of the diagnose, the state and municipal governments proposed several measures in minimizing the spread of the virus among the population.

In March, Brazil recorded 2,239 more deaths from respiratory failure and pneumonia than in the same month of the previous year^13^. The number of Brazilians hospitalized exceeded 40 thousand in the first half of April with respiratory symptoms, of which only 15% had a confirmed diagnosis for COVID-19^14^. In Italy, the epicenter of the disease in Europe, 2500, new ICU beds were needed to treat patients with respiratory failure, resulting from COVID-19^15^. In Sergipe, the average number of ICU beds per 10,000 inhabitants is approximately 0.9, almost three times less than recommended by the Ministry of Health. Due to the limitation of the health system for the considerable expansion of the number of ICU beds in the State of Sergipe and to minimize the burden on health services, control measures are justified^12^.

Authorities have been challenged to adopt effective public health measures to combat COVID-19. As long as there are no specific vaccines and therapeutic procedures, as well as more information about the means of transmission and the profile of asymptomatic patients, the momentary objective aims to contain the expansion of the virus to avoid overcrowding in health facilities. For this purpose, the social distancing was proposed seeking to reduce interaction, and perhaps transmission, between people in the community since there may be asymptomatic infected people transmitting the virus^16^. Based on these data, social distancing measures are justified, which initially included the closure of schools, gyms, clubs, and bars, the closure of part of the trade (deemed not essential), and the prohibition of parties and events, to avoid crowds.

Regarding the results obtained in this study, concerning the evolution of the pandemic in Sergipe, the number of coronavirus infections increased over time. It is also noticed that the adoption of isolation measures could be able to slow the growth of cases in the State. It is estimated that the pandemic could be contained in the second half, reaching less than half of infections if there is no more social isolation (a scenario in which all individuals would be infected sooner or later).

Based on previous experience in the management of SARS-CoV and MERs-CoV infections, WHO has recommended some control interventions to reduce the risk of transmission of acute respiratory infections. Including avoiding close contact between people with severe respiratory infections and washing hands frequently with soap and water or cleaning with 70% alcohol. Moreover, people with symptoms of acute respiratory disease should practice cough etiquette, which is, keeping distance, and covering their nose and mouth when coughing or sneezing with their forearms. As well as washing their hands within health facilities is recommended standard infection prevention and control practices, especially in emergency services^17^.

In a mathematical modeling study, developed by Koo and collaborators^18^, the impact of early interventions on the spread of COVID-19 in the population of Singapore was evaluated. Among the proposed interventions were (1) the isolation and quarantine of infected individuals and their families; (2) quarantine and immediate closure of schools for two weeks; (3) quarantine and social detachment from workers, with 50% of the workforce being encouraged to maintain work activities at home for two weeks; and (4) a combination of quarantine, with the immediate closure of schools and distance from the workplace. The researchers showed that the combined strategy of social distance measures increased the reduction of COVID-19 infection by 78.2% (IQR 59.0 - 94.4).

In this study, it is possible to observe the significant impact of social isolation measures since, with these measures, the new support capacity is reduced. Notably, the maximum expected number of infected people would be 1,171,353 cases, with the forecast for the end of August 2020. Since about 80% of these are asymptomatic, we would have 234,271 infected people in the State. Of these, 11,713 would need assistance in the intensive care unit, which represents 5% of those infected^7^_._

On the other hand, if isolation is neglected, there could be an infection of all individuals in the State, that is, about 2.3 million cases. Thus, new transmissions would be made available, especially in the most vulnerable part of the population. These are expected to be among the approximately 5% of those infected who would need treatment in the ICU^7^. However, the number of beds of this type in Sergipe is 177. Stricter control measures, such as lockdown, are thus justified to avoid the collapse of the local health system^12^. Knowing that the maximum number of infected individuals who will need hospitalization is about 40,265, scheduled for November, there would be time for the quantitative adaptation of the health system concerning the expansion of beds, in addition to the adequate dimensioning of health professionals.

Although the scientific basis for health interventions is robust, there are other ethical and economic considerations involved. Equally important, political leaders need to maintain policies of quarantine and social detachment without any bias regarding any population group^19^. These interventions can represent risks of income reduction and even job loss for many families, disproportionately affecting the most vulnerable populations. Therefore, policies to reduce these risks must be implemented urgently^20^.

The COVID-19 pandemic has become a threat to the general population and health workers worldwide in particular, and despite this, knowledge about the new virus remains limited. On the other hand, effective treatment options, as well as the development of a vaccine, are still being studied^21^ ^-^ ^23^. Therefore, the aggressive implementation of infection control measures is essential to prevent the spread of the virus through human-to-human transmission.

There is likely to be an influence of multiple indirect effects of interventions and changes in human behavior, which are difficult to quantify. With this model, it was not possible to infer the most appropriate times to implement each intervention, nor how for how long they should be carried out for the adequate reach of long-term epidemic control to be explored. This work does not deal directly with spatial diffusion nor consider the heterogeneity in the contact matrix. Furthermore, the data provided could be out of date due to the delay in delivery by the health departments and false-negative results. The voidness of the data could as well be due to the reduced availability of tests that applied in general, exclusively to symptomatic patients, which correspond to a lower than the actual number of infected.

Regarding current data, this study may assist the authorities in making decisions and implementing public policies in the short and medium-term in mitigating the virus among the population, and in the planning to create new beds for the treatment of the infected, whenever necessary.

With the COVID-19 biomathematical transmission model, used in the State of Sergipe, Brazil, it was possible to project its epidemic growth throughout the second half of 2020. From the estimates made, it is possible to contemplate, in different scenarios, the dimension of the impact of interventions on the size of the epidemic outbreak. Specifically, the use of lockdown is the control intervention with the most exceptional capacity to mitigate the spread of the virus, drastically reducing the number of cases, perhaps deaths, in the population.
